# Timing of Development of Symptomatic Brain Metastases from Non-Small Cell Lung Cancer: Impact on Symptoms, Treatment, and Survival in the Era of Molecular Treatments

**DOI:** 10.3390/cancers12123618

**Published:** 2020-12-03

**Authors:** Stephanie T. Jünger, Petra Schödel, Daniel Ruess, Maximilian Ruge, Julia-Sarita Brand, Maike Wittersheim, Marie-Lisa Eich, Nils-Ole Schmidt, Roland Goldbrunner, Stefan Grau, Martin Proescholdt

**Affiliations:** 1 Centre for Neurosurgery, Department of Neurosurgery, Faculty of Medicine and University Hospital, University of Cologne, 50931 Cologne, Germany; roland.goldbrunner@uk-koeln.de (R.G.); stefan.grau@uk-koeln.de (S.G.); 2 Centre for Integrated Oncology, Faculty of Medicine and University Hospital, University of Cologne, 50931 Cologne, Germany; daniel.ruess@uk-koeln.de (D.R.); maximilian.ruge@uk-koeln.de (M.R.); 3 Department of Neurosurgery, University Medical Centre Regensburg, 93053 Regensburg, Germany; Petra.Schoedel@klinik.uni-Regensburg.de (P.S.); Nils-Ole.Schmidt@klinik.uni-Regensburg.de (N.-O.S.); martin.proescholdt@gmail.com (M.P.); 4 Wilhelm Sander Neuro-Oncology Unit, University Medical Centre Regensburg, 93053 Regensburg, Germany; 5 Centre for Neurosurgery, Department of Stereotactic and Functional Neurosurgery, Faculty of Medicine and University Hospital, University of Cologne, 50931 Cologne, Germany; julia-sarita.brand@uk-koeln.de; 6 Department of Pathology, Faculty of Medicine and University Hospital, University of Cologne, 50931 Cologne, Germany; maike.wittersheim@uk-koeln.de (M.W.); marie-lisa.eich@uk-koeln.de (M.-L.E.)

**Keywords:** brain metastasis, radiosurgery, neurosurgery, precocious, synchronous, metachronous, genetic mutations

## Abstract

**Simple Summary:**

In order to clarify whether an early development of brain metastases from non-small cell lung cancer represents a poor prognostic factor for further survival we analyzed 377 patients with brain metastases, treated by radiosurgery or surgery at two German institutions. Our results show that an early appearance of brain metastasis does not influence further survival in a comprehensive treatment setting.

**Abstract:**

Objective: We attempted to analyze whether early presentation with brain metastases (BM) represents a poor prognostic factor in patients with non-small cell lung cancer (NSCLC), which should guide the treatment team towards less intensified therapy. Patients and methods: In a retrospective bi-centric analysis, we identified patients receiving surgical treatment for NSCLC BM. We collected demographic-, tumor-, and treatment-related parameters and analyzed their influence on further survival. Results: We included 377 patients. Development of BM was precocious in 99 (26.3%), synchronous in 152 (40.3%), and metachronous in 126 (33.4%) patients. The groups were comparable in terms of age (*p* = 0.76) and number of metastases (*p* = 0.11), and histology (*p* = 0.1); however, mutational status significantly differed (*p* = 0.002). The precocious group showed the worst clinical status as assessed by Karnofsky performance score (KPS) upon presentation (*p* < 0.0001). Resection followed by postoperative radiotherapy was the predominant treatment modality for precocious BM, while in syn- and metachronous BM surgical and radio-surgical treatment was balanced. Overall survival (OS) did not differ between the groups (*p* = 0.76). A good postoperative clinical status (KPS ≥ 70) and the application of any kind of adjuvant systemic therapy were independent predictive factors for OS. Conclusion: Early BM presentation was not associated with worse OS in NSCLC BM patients.

## 1. Introduction

Lung cancers represent the most frequent cancer type seeding to the brain [1,2]. While some patients may develop brain metastases (BM) during or after initial treatment for the primary tumors, others show early development of brain disease, sometimes even before the primary cancer is detected. In accordance, the pattern of appearance is termed synchronous (i.e., within three months of diagnosis) or metachronous (i.e., later than three months after diagnosis) and precocious (i.e., prior to diagnosis of the primary tumor) [3,4,5].

While precocious and most synchronous metastases are frequently treatment-naive compared to metachronous tumors, a different oncological course for these patients has been reported, with a rather dismal prognosis for patients with precocious tumors, possibly resulting in differing treatment decisions [6].

As (radio-)oncological treatment patterns have changed substantially over recent decades due to the substances available and frequency of treatment, and with postoperative treatment options advancing over the past two decades [7], the aim of this study was to evaluate the treatment course of patients with symptomatic brain metastases from non-small cell lung cancers (NSCLC) in relation to their time of appearance in the setting of comprehensive oncological treatment.

## 2. Results

### 2.1. Demographic and Baseline Clinical Data

We identified 438 patients, who received treatment for brain metastases from lung cancer between 2010 and 2019. Sixty-one patients were excluded due to small cell histology, missing data, or postsurgical in-house mortality, leaving 377 patients in the analysis.

Histology comprised adeno-carcinoma (*n* = 309; 82%), squamous cell carcinoma (*n* = 46; 12.2%), neuro-endocrine differentiation (*n* = 17; 4.5%), and not otherwise specified (*n* = 5; 1.3%) and was not statistically different with regard to BM timing (*p* = 0.10).

The median age was 61 (range 25–87) years, of which 207 (54.9%) patients were male. Median timespan since initial tumor diagnosis was 1.8 (range −4–162) months, resulting in 99 (26.3%) precocious, 152 (40.3%) synchronous, and 126 (33.4%) metachronous BM.

Distribution of age (*p* = 0.76) and number of metastases (*p* = 0.11) were similar between these groups, while the pattern of driver mutations was different (*p* < 0.0001; Table 1). Patients with precocious BM presented significantly more frequently with neurological symptoms (*p* < 0.0001) and had a worse clinical status than patients with synchronous and metachronous BM (*p* = 0.03).

Detailed information regarding tumor location and symptoms is displayed in Table 1.

### 2.2. Treatment of BM

For treatment of BM, 132 patients underwent Cyberknife^®^ stereotactic radiosurgery (SRS), while 245 patients underwent resection of at least one BM followed by radiotherapy.

Patients receiving surgical resection presented more frequently with precocious metastases (*p* = 0.002) and had a significantly higher symptom burden (*p* < 0.0001), whereas the patients treated with radiosurgery displayed significantly more oligo- and multiple metastases (*p* < 0.001).

### 2.3. Driver Mutation Status

Molecular analysis of tumor tissue was available for 235 patients; the frequency of mutations did significantly differ between precocious, synchronous, and metachronous BM (Table 1); however, a distinct and characteristic pattern was not detected.

### 2.4. Clinical Status

The median Karnofsky performance scale (KPS) of the entire cohort improved from a score of 80 (40–100) before, to 90 (40–100) after treatment. Accordingly, the radiation therapy oncology group (RTOG) recursive partitioning analysis (RPA) class of surgically treated patients improved from class III to classes I or II for 34 patients after BM treatment due to an improvement in postoperative symptoms, which resulted in an improved KPS. In four patients, neurological deterioration resulted in a poorer class after BM resection.

Systemic treatment after BM therapy was administered to 241 patients during their further disease course, with a tendency for precocious and synchronous tumors (*p* = 0.09) to be more frequent.

The frequency of systemic treatment after BM therapy varied substantially during the study period with a clear increase over time (e.g., first vs. second half of the study period, 57 vs. 76%; *p* = 0.002). Detailed medical data were available for 238 patients and are displayed in Table 1.

### 2.5. Survival Outcome

By the time of analysis, 232 (61.5%) patients had died. Among the known causes of death, systemic disease progression was the most prevalent in all groups. Median overall survival was 14.1 months (95% CI 12.2–15.8).

**Figure 1:**
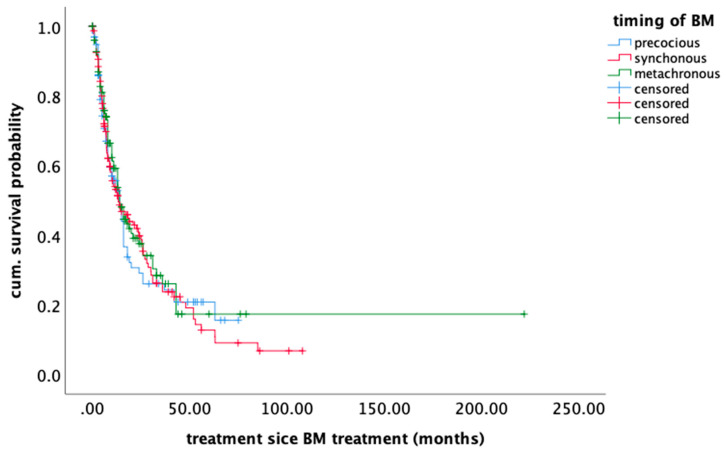
Kaplan–Meier plot showing overall survival of patients bearing brain metastases depending on the time of appearance.

In the univariate analysis, the timing of BM development did not influence further survival (*p* = 0.76) (Figure 1), whereas the clinical status (KPS) after BM treatment (*p* < 0.0001), postoperative systemic treatment (*p* < 0.0001), and treatment modality—surgical vs. radio-surgical—(*p* = 0.002) did influence survival. Age ≥ 65 (*p* = 0.88), systemic disease control (*p* = 0.15), and number of BM (*p* = 0.99) did not have a significant impact on OS. (Table 2)

In multivariate regression, only the post-therapeutic clinical status (hazard ratio (HR) 0.38 0.25–0.58 *p* < 0.0001) and systemic therapy (HR 0.48 0.37–0.63 *p* < 0.0001) remained as independent prognostic factors. (Table 2)

## 3. Discussion

In cancer patients, the impact of the timespan until they develop BM on their further survival is unclear. Only a few studies are dedicated to this topic [3,8,9,10], and even fewer compare the actual points in time to one another [6,10].

In the present study, survival did not differ between precocious, synchronous, and metachronous patients if they received tumor-specific therapy after local brain metastases treatment was completed. Our findings are in contrast to data from Shibahara et al., Thomas et al., and Wronski et al., who observed a particularly poor prognosis in patients developing early brain disease [3,6,11].

Contradicting results were reported for patients who were treated for primary disease and BM simultaneously, showing no survival correlation with the time of BM occurrence, but instead with the extent of primary tumor resection, as well as the status of lymph-nodular metastasis [12,13,14,15,16]. However, most of these studies were conducted in a pre-radio-surgical era and when targeted treatment options were not yet available. Thus, it must be emphasized that the present study mainly reports on a cohort of patients treated in the modern era with access to available targeted therapies and radio-surgical techniques.

Therefore, a major difference in the present cohort may be the high percentage of systemic treatments after BM therapy, previously reported as a positive prognostic factor [10]. In particular, patients with precocious and synchronous tumors received systemic treatment due to a previously untreated primary disease. This reflects the change in paradigms which has taken place within the last few decades: the appearance of BM is no longer a substantial reason for a treatment decision towards local measures such as whole brain radiation therapy (WBRT) with a palliative intention, but is rather one step in a comprehensive oncological treatment concept including novel molecular substances crossing the blood–brain barrier. Since the largest number of treatable driver mutations have been discovered for lung cancers, subsequent effective therapy after BM treatment is available to a larger extent than for most other tumors [17,18,19,20], and the discordance rate between primary tumor and BM has been reported as low [21]. This is reflected by the high percentage of systemic treatments after BM therapy and the increase in such treatments over time in this present cohort, which was observed to be independent from the timing of BM development.

In the present study, we therefore focused on non-small cell lung cancer as a particular tumor entity in order to generate more specific conclusions facilitated by a more homogenous study population. Lung cancer is among the tumors to most frequently spread to the brain and is the origin of the majority of precocious and synchronous metastases [3,11]. This may be explained by: (a) the high tendency of these cancers themselves to spread to the brain; (b) a portion of the population prone to this cancer type frequently being heavy smokers, who may tend to neglect physical symptoms for a longer time; and (c) the increasing inclusion of cerebral screening after radiologically suspected lung cancer.

The significantly higher symptomatic burden and impaired clinical performance status of patients with precocious BM may explain the predominance of these patients in the surgical subgroup. This is mirrored by the striking differences in neurological symptom prevalence and clinical performance status between the treatment groups. While the latter is a key aspect in prognostic grading scores [22,23], surgery may influence the overall prognosis rather indirectly by improving the clinical status, underlined in this present study by pre- and postoperatively different RPA group allocation. In this context, the different statistical effect of the treatment modality on overall survival can be well explained: while radiosurgery showed a univariate survival benefit compared to the surgical group, this effect was no longer seen after adjustment for the postoperative clinical status in multivariate regression.

This observed clinical benefit seen in the improvement of neurological symptoms in the surgical group is essential, as the patient’s clinical status is established to aid treatment decision-making. Consequently, resection of a symptomatic brain mass may enable successful further treatment, while for asymptomatic patients, a less invasive treatment regime such as radiosurgery appears to be the preferred option with equal value [24].

This study carries all the limitations and biases of a retrospective design. Besides the intrinsic heterogeneity of the population due to different clinical and molecular statuses, the treatment paradigms and settings also changed within the study period. Apart from the technical inventions making brain tumor surgery safer (such as intraoperative monitoring), radio-oncological techniques have also been adjusted from WBRT towards more local approaches (such as radiosurgery and focal radiotherapy). Furthermore, oncological attitudes towards an ongoing and comprehensive treatment after BM diagnosis have changed substantially with respect to available substances. These changes are reflected in the increasing number of (novel) treatments over time.

Therefore, these data may not support a benefit from any particular systemic treatment after BM, but rather should answer the question of whether further treatment after BM should depend on the timing at all.

## 4. Patients and Methods

The study was an institutional retrospective study approved by the local ethics committees (University of Cologne approval no. 18-089; University of Regensburg approval no. 15-101-0065).

We queried our institutional databases for adult patients who underwent surgery and stereotactic radiosurgery (SRS) for BM from lung cancer between 2010 and 2019 and identified demographic and clinical parameters.

The time to diagnosis of BM was calculated from the date of primary tumor diagnosis, until the date of diagnosis of BM. On the basis of the time span between initial diagnosis and BM, we allocated the patients to three groups using a previously reported classification: (1) precocious (BM diagnosed as a first sign of disease, before any primary tumor was detected); (2) synchronous (BM diagnosed within three months after diagnosis of the primary tumor. Diagnosis was made using neurological symptoms or standard staging work-up); and (3) metachronous (BM appeared later than three months after initial cancer diagnosis). Postsurgical survival was calculated from the date of BM resection, until death or last follow-up.

Patients were allocated to RTOG recursive partitioning analysis classes [22,25].

All data for the Cologne cohort were collected from electronic and paper charts and documented using a REDCap database.

Treatment decisions were reached within an interdisciplinary institutional tumor board, involving board-certified neurosurgeons, neuro-oncologists, medical oncologists, neuro-radiologists, neuropathologists, and palliative care physicians.

SRS was carried out with a robotic-guided linear accelerator (LINAC) (Cyberknife ^®^, Sunnyvale, CA, USA). For treatment planning, all BM were routinely outlined on contrast enhanced, T1-weighted MRI (Phillips, MR-Scanner 1.5 or 3 Tesla), which was obtained a few days prior to SRS and registered to a stereotactic planning computer tomography (CT) (1 mm slice thickness, Phillips 8-slice or 16-slice multidetector CT). For treatment, the patient was immobilized with a custom-made aquaplast mask. The final irradiation plan was evaluated in an interdisciplinary consensus meeting between the stereotactic neurosurgeon, a radiation oncologist experienced in SRS, and the medical physicist.

The dose subscribed to the surface of the BM ranged between 18 and 20 Gy (65% isodoses level) depending on previous irradiation.

Surgery was performed under general anesthesia. If required, the procedure was conducted using intraoperative optic navigation, and in the case of eloquent location, cortex stimulation. The postoperative extent of resection was assessed by cMRI within 24 to 48 h.

For mutational analysis, a representative tumor era was selected by a board-certified pathologist or neuropathologist. As previously described, isolated DNA was amplified with a customized lung panel (nNGM Panel 1.0) and the GeneRead DNAseq Panel PCR Kit V2 (Qiagen, Hilden, Germany), or an Ion AmpliSeq Library Kit (LifeTechnologies, Thermo Fischer Scientific, Carlsbad, CA, USA) according to manufacturers’ protocol. Libraries were constructed using the Gene Read DNA Library I Core Kit and the Gene Read DNA I Amp Kit (Qiagen). For adapter ligation, the NEXTflex-96 DNA Barcodes (Bioo Scientific, Austin, TX, USA) were used. Library products were quantified with the Qubit dsDNA HS Assay Kit (Thermo Fisher Scientific, Waltham, MA, USA) on the Qubit 2.0 Fluorometer (Thermo Fisher Scientific, Waltham, MA, USA), diluted, and pooled in equal amounts. A total of 12 pM was sequenced on the MiSeq (Illumina, San Diego, CA, USA) with a MiSeq reagent Kit V2 (300-cycles) (Illumina). Data were exported as FASTQ files. Alignment and annotation were performed using a modified version of a previously described method [26] The resulting BAM files were visualized using the Integrative Genomics Viewer (IGV; http://www.broadinstitute.org/igv/, Cambridge; USA). A 5% cutoff for variant calls was used, and results were only interpreted if the coverage was >200×.

FISH (Fluorescence in situ hybridization) was performed as previously described [27] using commercially available FISH probes provided by Zytovision (ZytoVision GmbH, Bremerhaven, Germany) according to manufacturer’s instructions. FISH for MET amplification, ROS1 translocation, and RET translocation were performed at the time of diagnosis. ALK (anaplastic lymphoma kinase) translocation FISH was performed in case of any positive or ambiguous immunohistochemistry result. Slides were reviewed at high magnification (×63) and scored according to the appropriate, respective guidelines.

Statistical analysis was performed using SPSS Statistics Version 25 (IBM, Armonk, NY, USA). For descriptive statistics, continuous values are given in median and range; ordinal and categorical variables are stated in counts and percentage. A Chi-squared test and Kruskal–Wallis test were used to identify correlations between parameters. Survival rates were estimated using the Kaplan–Meier method. Univariate analysis (Log-rank test) was used to identify covariates with an impact on survival after BM resection. Multivariate Cox regression was conducted for significant factors in univariate analysis using the pairwise Inclusion Method. *P*-values lower than 0.05 were considered statistically significant.

## 5. Conclusions

Although the development of BM may initiate the final stage of the disease for most patients, the timing of BM development in patients with NSCLC treated in an interdisciplinary setting does not influence their further clinical course. Thus, patients with early brain metastases benefit from systemic treatment regarding survival.

## Figures and Tables

**Table 1:** Demographics and baseline characteristics.

Parameter	Precocious (*n* = 99)	Synchronous (*n* = 152)	Metachronous (*n* = 126)	All (*n* = 377)	*p*-Value
Age [median; range]	62 (38–87)	62 (32–85)	60 (25–83)	61 (25–87)	0.76
Male gender [*n*;%]	56 (56.6)	88 (57.9)	63 (50.0)	207 (54.9)	0.26
Controlled primary disease [*n*;(%)]	0 (0)	37 (24.3)	57 (45.2)	94 (24.9)	<0.0001
Histology [*n*;%]					0.10
Adeno	78 (78.8)	127 (83.5)	104 (82.5)	309 (82.0)	
Squamous cell	14 (14.1)	13 (8.6)	19 (15.1)	46 (12.2)	
Neuro-endocrine	0 (0)	3 (2.0)	2 (1.6)	5 (1.3)	
NOS	7 (7.1)	9 (5.9)	1 (0.8)	17 (4.5)	
Previous treatment for primary disease					<0.0001
none	99 (100)	16 (10.5)	4 (3.2)	119	
Surgery	0 (0)	27 (17.8)	68 (54.0)	95	
Neo-adjuvant Chemotherapy	0 (0)	14 (9.2)	20 (15.9)	34	
Neo-adjuvant radiotherapy	0 (0)	0 (0)	12 (9.5)	12	
Chemotherapy	0 (0)	63 (41.4)	89 (70.6)	152	
Radiotherapy	0 (0)	21 (13.8)	47 (37.3)	68	
Molecular treatment	0 (0)	6 (3.9)	26 (20.6)	32	
Unknown	0 (0)	5 (3.3)	5 (4.0)	10	
BM count [*n*;%]					0.11
1 BM	60 (60.6)	80 (52.6)	59 (46.8)	199 (52.8)	
2–3 BM	29 (29.3)	51 (33.6)	38 (30.2)	118 (31.3)	
≥4 BM	10 (10.1)	30 (19.8)	29 (23.0)	69 (18.3)	
Mutational status [*n*;%]					0.002
N/A	29 (29.3)	79 (52.0)	34 (27.0)	142 (37.7)	
Wild type	36 (36.4)	18 (11.8)	40 (31.7)	94 (24.9)	
EGFR	4 (4.0)	28 (18.4)	19 (15.1)	51 (13.6)	
KRAS	7 (17.2)	20 (13.2)	21(16.7)	58 (15.4)	
MET	6 (6.1)	3 (2.0)	4 (3.2)	13 (3.4)	
BRAF	1 (1.0)	0 (0)	1 (0.8)	2 (0.5)	
ALK	1 (1.0)	1 (0.7)	1 (0.8)	3 (0.8)	
ROS	0 (0)	0 (0)	3 (2.4)	3 (0.8)	
FGFR	2 (2.0)	1 (0.7)	1 (0.8)	4 (1.1)	
PIK3CA	0 (0)	2 (1.3)	0 (0)	2 (0.5)	
ErbB2	3 (3.0)	0 (0)	2 (1.6)	5 (1.3)	
Tumor location [*n*;(%)]					0.17
Supratentorial	46 (46.5)	86 (56.6)	64 (50.8)	196 (52.0)	
Infratentorial	22 (22.2)	25 (16.4)	11 (8.7)	58 (15.4)	
Supra- and infratentorial	31 (31.3)	41 (27.0)	51 (40.5)	123 (32.6)	
Symptomatic BM [*n*;(%)]					<0.0001
Neurological deficits [*n*]	97 (98.0)	90 (59.2)	71 (56.3)	258 (68.4)	
Seizures	15	21	12	48	
Aphasia	14	11	8	33	
Hemiparesis	26	28	21	75	
Visual field defects	1	13	5	19	
Cerebellar signs	39	10	20	69	
Signs of elevated intracranial pressure	30	26	18	74	
KPS at presentation [median; range]	80 (40–100)	90 (40–100)	90 (50–100)	80 (40–100)	0.03
RPA class prior to BM treatment					<0.0001
Class I	0	0	34	34	
Class II	79	133	72	284	
Class III	20	19	20	59	
RPA class after BM treatment					<0.0001
Class I	0	1	34	35	
Class II	97	139	78	314	
Class III	2	12	14	28	
Treatment modality					<0.0001
Surgery + postoperative radiotherapy	84 (84.8)	91 (59.9)	70 (55.6)	245 (65.0)	
Stereotactic radiosurgery	15 (15.2)	61 (40.1)	56 (44.4)	132 (35.0)	
Systemic medical treatment after BM					0.09
Treatment modality (*n* = 238)	68 (68.7)	102 (67.1)	71 (56.3)	241 (63.9)	
Chemotherapy	42	72	57	171	
Molecular therapy	39	39	24	102	
Dead by time of analysis	62 (62.6)	103 (67.8)	67 (53.2)	232 (61.5)	
Cause of death					<0.0001
Unknown	8 (12.9)	67 (65.0)	23 (34.3)	101 (43.5)	
Neurological	4 (6.5)	7 (6.8)	10 (14.9)	21(9.1)	
Systemic disease progression	46 (74.1)	26 (25.2)	34 (50.7)	106 (45.7)	
others	4 (6.5)	3 (2.9)	6 (9.0)	13 (5.6)	

Abbreviations used in Table 1: ALK (anaplastic lymphoma kinase), BM (Brain metastasis/es), BRAF (v-raf murine sarcoma viral oncogene homolog B1), EGFR (epidermal growth factor recetor), ErbB2 (Erb-B2 Receptor Tyrosine Kinase 2), FGFR (fibroblast growth factor receptor), KPS (Karnofsky Performance Status), KRAS (Kirsten rat sarcoma viral oncogene), MET (Mesenchymal–epithelial transition), N/A (not applicable), PIK3CA (Phosphatidylinositol-4,5-Bisphosphate 3-Kinase Catalytic Subunit Alpha), ROS (reactive oxygen species), RPA (recursive partitioning analysis).

**Table 2:** Analysis of prognostic factors in uni- and multivariate analysis.

Parameter	Univariate (Log Rank) [*p*-Value]	Multivariate (Cox Regression) [HR 95%CI; *p*-Value]
Age ≤ 65	0.88	
Controlled systemic status	0.15	
Timing	0.76	
Precocious vs. synchronous	0.71	
Precocious vs. metachronous	0.82	
Synchronous vs. metachronous	0.33	
KPS ≥ 70 post-BM-treatment	<0.0001	0.38, 0.28–0.58, *p* < 0.0001
BM count		
Single vs. oligo vs. multi	0.99	
Systemic treatment after BM	<0.0001	0.48, 0.39–0.63, *p* < 0.0001
Radiosurgery only	0.002	n.s.

## Abbreviations

ALK	anaplastic lymphoma kinase
BM	brain metastasis
BRAF	v-raf murine sarcoma viral oncogene homolog B1
CT	computer tomography
EGFR	epidermal growth factor recetor
ErbB2	Erb-B2 Receptor Tyrosine Kinase 2
FGFR	fibroblast growth factor receptor
HR	Hazard ratio
KPS	Karnofsky performance scale
KRAS	Kirsten rat sarcoma viral oncogene
LINAC	linear accelerator
MET	Mesenchymal–epithelial transition
(c)MRI	(cranial)magnetic resonance imaging
N/A	not applicable
NSCLC	non-small cell lung cancers
OS	overall survival
PIK3CA	Phosphatidylinositol-4,5-Bisphosphate 3-Kinase Catalytic Subunit Alpha
ROS	(reactive oxygen species
RPA	recursive partitioning analysis
RTOG	Radiation Therapy Oncology Group
SRS	stereotactic radiosurgery
WBRT	whole brain radiation therapy
